# Analysis of the Characteristics and Sources of Carbonaceous Aerosols in PM_2.5_ in the Beijing, Tianjin, and Langfang Region, China

**DOI:** 10.3390/ijerph15071483

**Published:** 2018-07-13

**Authors:** Mengxi Qi, Lei Jiang, Yixuan Liu, Qiulin Xiong, Chunyuan Sun, Xing Li, Wenji Zhao, Xingchuan Yang

**Affiliations:** 1 Beijing Key Laboratory of Resources Environment and Geographic Information System, Capital Normal University, Beijing 100048, China; 2160902114@cnu.edu.cn (M.Q.); 2160902137@cnu.edu.cn (Y.L.); 2140901012@cnu.edu.cn (Q.X.); 2140902114@cnu.edu.cn (C.S.); 2160902100@cnu.edu.cn (X.L.); 2160902148@cnu.edu.cn (X.Y.); 2 Base of the State Key Laboratory of Urban Environmental Process and Digital Modelling, Beijing 100048, China; 3 Beijing Municipal Research Institute of Environmental Protection, Beijing 100037, China; jiangle3657@sina.com

**Keywords:** carbonaceous aerosols, PM_2.5_, organic carbon, elemental carbon, source analyses

## Abstract

PM_2.5_ samples from Beijing, Tianjin, and Langfang were simultaneously collected from 20 November 2016 to 25 December 2016, and the organic carbon (OC) and elemental carbon (EC) content in the samples were measured and analyzed. The pollution characteristics and sources of OC and EC in atmospheric PM_2.5_ for three adjacent cities were discussed. The average mass concentrations of OC in PM_2.5_ in Beijing, Tianjin, and Langfang were 27.93 ± 23.35 μg/m^3^, 25.27 ± 12.43 μg/m^3^, and 52.75 ± 37.97 μg/m^3^, respectively, and the mean mass concentrations of EC were 6.61 ± 5.13 μg/m^3^, 6.14 ± 2.84 μg/m^3^, and 12.06 ± 6.81 μg/m^3^, respectively. The average mass concentration of total carbon (TC) accounted for 30.5%, 24.8%, and 49% of the average mass concentration of PM_2.5_ in the atmosphere. The total carbonaceous matter (TCA) in Beijing, Tianjin, and Langfang was 51.29, 46.57, and 96.45 μg/m^3^, respectively. The TCA was the main component of PM_2.5_ in the region. The correlation between OC and EC in the three cities showed R^2^ values of 0.882, 0.633, and 0.784 for Beijing, Tianjin, and Langfang, respectively, indicating that the sources of urban carbonaceous aerosols had good consistency and stability. The OC/EC values of the three sampling points were 4.48 ± 1.45, 4.42 ± 1.77, and 4.22 ± 1.29, respectively, considerably greater than 2, indicating that the main sources of pollution were automobile exhaust, and the combustion of coal and biomass. The OC/EC minimum ratio method was used to estimate the secondary organic carbon (SOC) content in Beijing, Tianjin and Langfang. Their values were 10.73, 10.71, and 19.51, respectively, which accounted for 38%, 42%, and 37% of the average OC concentration in each city, respectively. The analysis of the eight carbon components showed that the main sources of pollutants in Beijing, Tianjin, and Langfang were exhaust emissions from gasoline vehicles, but the combustion of coal and biomass was relatively low. The pollution of road dust was more serious in Tianjin than in Beijing and Langfang. The contribution of biomass burning and coal-burning pollution sources to atmospheric carbon aerosols in Langfang was more prominent than that of Beijing and Tianjin.

## 1. Introduction

Carbonaceous aerosols are an important part of atmospheric fine particulate matter (PM_2.5_) in China and have a considerable impact on the atmospheric environment [1], accounting for approximately 20–50% of the PM_2.5_ mass concentration [2,3,4,5,6,7]. Carbonaceous aerosols are composed of organic carbon (OC) and elemental carbon (EC) [8]. The OC is mainly composed of primary organic carbon (POC) and secondary organic carbon (SOC), which is produced by photochemical reactions from pollutants. The components of OC are very complex and rich in toxic substances, and have the potential to cause great harm to human health [9,10]. The EC is also known as black carbon by most domestic and foreign scholars and is mainly derived from the incomplete combustion of carbonaceous matter. Because EC has the characteristic of absorbing solar radiation, it plays an important role in promoting global warming, and it is also one of the main triggers of reduced atmospheric visibility [11,12,13].

In recent years, with the increasingly severe situation of regional air pollution in China, domestic and foreign scholars have carried out a considerable amount of research on carbonaceous aerosols. For example, related researchers [2,14] studied the change characteristics of OC and EC of PM_2.5_ in Beijing and found that the concentration of carbonaceous organic matter was higher in winter than in summer, higher at night than in the daytime, and high in the south and low in the north of China. Ji et al. [15] conducted a comparative analysis of the variation characteristics of carbon-bearing aerosols in Beijing from 2013 to 2014. It was found that the annual mean value of carbonaceous aerosols in the atmosphere was similar between these two years, but as time passed, the effect of the EC in the atmosphere increased gradually and the contribution of traffic sources to air pollution has become increasingly serious. An atmospheric PM_2.5_ and PM_10_ study found that the content of carbonaceous aerosols is high in the Tianjin atmosphere, and coal combustion and motor vehicle exhausts are the main sources of atmospheric particulate pollution during the winter [16]. Based on the analysis of the carbon composition of PM_2.5_ during the heating period in Tianjin from 2009 to 2010, Huo et al. [17] found that the pollution of atmospheric carbonaceous aerosols in Tianjin mainly comes from biomass combustion, automobile exhaust gas, coal burning, and road dust. Zhou et al. [18] compared the atmospheric samples between haze episodes and clear day and concluded that the accumulation effect of carbonaceous organic matter during the pollution process was obvious under the haze weather conditions, and the formation of secondary substances in carbonaceous aerosols was also apparent. Many scholars [19,20,21] have studied the Beijing–Tianjin–Hebei region at the regional level and found that the pollution concentrations of OC and EC in autumn and winter are generally higher than those in spring and summer because of the stable atmospheric pressure and low temperature. The average concentration of Baoding and Shijiazhuang in Hebei Province is higher than that of Beijing and Tianjin.

At present, there are many studies on carbonaceous aerosols in a single city or block area; to our knowledge, no studies have compared and analyzed the pollution characteristics and source differences of carbonaceous aerosols in the atmospheric PM_2.5_ of three neighboring cities (Beijing, Tianjin, and Langfang). Therefore, samples of PM_2.5_ were collected at the same time from the atmosphere in Beijing, Tianjin, and Langfang in the heating period. The differences in the pollution characteristics and sources of the carbonaceous aerosols in PM_2.5_ samples from the three cities were compared based on analysis of the changes of the OC and EC concentration. The results of this study could be used to provide scientific guidance and suggestions for the combined treatment of the atmospheric environment in Beijing, Tianjin, and Langfang.

## 2. Sampling and Analysis Method

### 2.1. Study Area and Sampling Site

As an important part of “Beijing–Tianjin–Hebei integration”, Beijing, Tianjin, and Langfang have played an important role in implementing the transportation and environment integration strategic of the region. Among them, Tianjin relies on its geographical advantages of being near the coast and has developed rapidly, and Langfang has strengthened the communication and cooperation among the three cities. The increasing environmental pollution pressure has caused city development to face an increasing amount of challenges. Air pollution in this region has a complex regional transmission and the typical characteristics of urban development under the control of the joint air pollution control policy between Beijing, Tianjin, and Hebei. It is therefore of great strategic value to examine the similarities and differences in the characteristics of air pollution on the development axis of Beijing, Tianjin, and Langfang, and determine pollution characteristics that can guide air pollution control across the whole Beijing–Tianjin–Hebei region.

**Figure 1:**
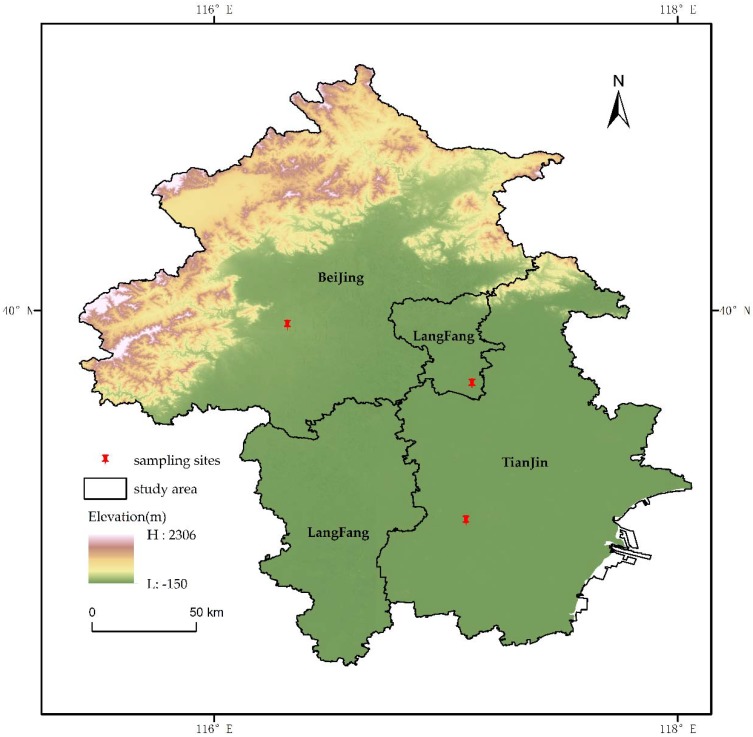
Locations of the study area and sampling sites.

Three sampling points were established in this study. The Beijing sampling point was positioned on the fourth floor of the teaching building of Capital Normal University, Beijing (116°18′ E, 39°55′ N), near the West Third Ring Road at about 15 m from the ground. It is surrounded by ordinary office buildings, schools, and residential areas. The Tianjin sampling point was the fourth floor of the Tianjin City Construction University (117°5′ E, 39°5′ N), on the west side of the campus at about 15 m from the ground. The sampling point is about 500 m away from the city’s arterial road and is adjacent to another branch road, surrounded by a large shopping mall and a large-scale residential area. The Langfang sampling point was in Xianghe County, Langfang, Hebei Province (117°6′ E, 39°40′ N) in an open space 5 m away from the residential area, about 2 m from the ground. There are no large-scale residential areas with centralized heating around the sampling site, and almost all of them are separate courtyards. The residents’ main heating method is self-sufficiency. The sampling site locations are shown in Figure 1. The data of the map comes from the city borders of 1:25 million standard geographic data in China.

### 2.2. Sample Collection and Chemical Analysis

Using a quartz filter membrane (Whatman, UK) and a large flow-rate sampling instrument (1.13 m^3^·min^−1^; TE-6070DV, Tisch, Medford, MA, USA) in Beijing and the same type of low flow rate sampling instrument (0.0167 m^3^·min^−1^; PQ200, BGI, Cambridge, MA, USA) in Tianjin and Langfang, PM_2.5_ particles were collected from the atmosphere from 20 November to 25 December 2016. The sampling time was set to 24 h per day from 8:00 a.m. one day to 8:00 a.m. the next day. After the removal of human-induced sample pollution (such as human error in the sampling process caused by damage to the quartz membrane) and instrument maintenance shutdown (1–4, 6, 9, and 23 December 2016), a total of 75 samples were obtained. Before sampling, the filter membrane was placed in a muffle furnace and baked for 5 h at 550 °C to remove the residual organic matter and impurities in the filter membrane. After the filtering membrane cooled, it was placed in a constant temperature humidity chamber to be balanced for 48 h. Finally, an electronic microbalance was used to weigh the treated filter membrane and the processed filter membrane was wrapped up with burned aluminum foil to prevent secondary pollution during use. The filter membranes were stored at −20 °C before and after sampling.

Using the Model 2001A thermal/optical carbon analyzer (American Desert Research Institute, Paradise, NV, USA), and following the IMPROVE (Interagency Monitoring of Protected Visual Environments) thermal/optical reflectance (TOR) protocol, the contents of the carbonaceous components in the quartz filter membrane were measured after sampling. The 0.529 cm^2^ sampling membranes were heated and analyzed in an oxygen-free environment of pure helium. According to the heating sequence of 120 °C, 250 °C, 450 °C, and 550 °C set by the instrument program, the component contents of OC1, OC2, OC3, and OC4 were analyzed, respectively, during which the organic matter in the particulate matter was completely converted into CO_2_. Then oxygen was introduced into the instrument to form an environment containing 2% oxygen and 98% helium, and then the temperature was sequentially increased to 550 °C, 700 °C, and 800 °C, to measure the content of EC1, EC2, and EC3 in the filter membrane, respectively. The EC in the particles was converted into CO_2_ to be released in the process. The resulting CO_2_ entered a reducing atmosphere, catalyzed by MnO_2_, and was reduced to CH_4_, which can be detected by a flame ionization detector (FID) under conditions of an ascending gradient temperature. In addition, some OC was carbonized and transformed into pyrolytic carbon (OP), which makes it difficult to distinguish between OC and EC. Therefore, the reflected light of the quartz film was monitored by a 633 nm He-Ne laser to determinate the demarcation point between OC and EC.

### 2.3. Quality Control

When weighing with an electronic microbalance, each quartz filter membrane was weighed twice and the average of the two results represented the final weight of the quartz filter membrane. Besides that, before and after sample analysis, it was necessary to calibrate the instrument (thermal/optical carbon analyzer) with CH_4_/CO_2_ standard gas. Furthermore, in order to ensure the accuracy of the measurement results, one out of every seven samples was randomly selected for a second analysis. The error between the two analysis results was less than 10% before the follow-up experiment was carried out, and the standard sample was tested every 3 days.

## 3. Results and Discussions

### 3.1. OC, EC, and PM_2.5_ Mass Concentration Characteristics

**Figure 2:**
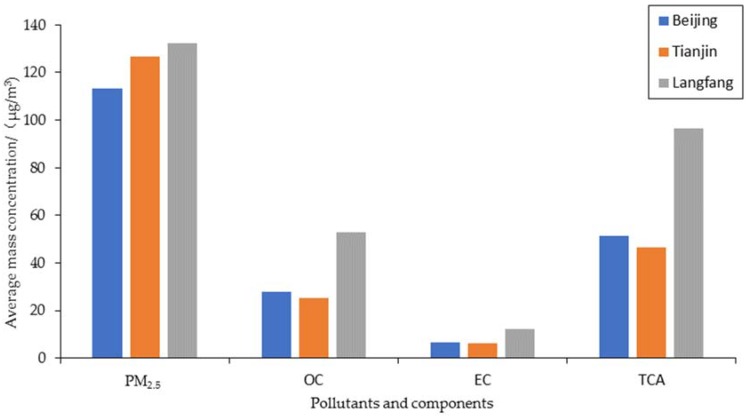
Average mass concentration of PM_2.5_, organic carbon (OC), and elemental carbon (EC) at the three sample sites.

The average mass concentrations of atmospheric PM_2.5_ and its carbonaceous chemical components (OC and EC) for Beijing, Tianjin, and Langfang from 20 November to 25 December 2016, are shown in Figure 2. Samples were collected from these three cities during the winter heating period. The average mass concentrations of atmospheric PM_2.5_ were 113.13 ± 93.03, 126.87 ± 79.10, and 132.16 ± 95.5 μg/m^3^, respectively. These values were higher than the daily average thickness limit value of the secondary grade standard of air pollution (75 μg/m^3^), which shows that PM_2.5_ pollution in the study area was serious. Moreover, the pollution levels of PM_2.5_ in the three cities increased in turn. The total carbon (TC) content accounted for 31%, 25%, and 49% of the PM_2.5_, respectively. The average concentration of atmospheric PM_2.5_ and TC showed the spatial distribution of Langfang > Tianjin > Beijing.

The average mass concentrations of OC and EC were 27.93 ± 23.35 μg/m^3^ and 6.61 ± 5.13 μg/m^3^ (Beijing), 25.27 ± 12.43 μg/m^3^ and 6.14 ± 2.84 μg/m^3^ (Tianjin), and 52.75 ± 37.97 μg/m^3^ and (12.06 ± 6.81) μg/m^3^ (Langfang), respectively, which presents a spatial characteristic of Langfang > Beijing > Tianjin. Tianjin and Beijing had similar concentrations of both OC and EC, and were considerably lower than Langfang, which may be related to the implementation of a change from burning coal to burning natural gas and a coal modification policy in Beijing and Tianjin. Compared with coal burning, the content of pollutants brought about by natural gas and power resources has been greatly reduced. In addition, because urban air quality is easily affected by air mass transport from different directions, the pollution level of Langfang is quite different from that of Beijing and Tianjin [16].

The total carbonaceous matter content (TCA) in the atmosphere is the sum of organic aerosol (OA) and EC. Some studies [22] defined the conversion factor between OA and OC as the average ratio of molecular weight of organic compounds to the molecular weight of carbon in these organic compounds, and this factor is 1.6 ± 0.2 in urban atmospheric aerosol studies, with which the best prediction effect is usually obtained [23]. The TCA in Beijing, Tianjin, and Langfang accounted for 45%, 37%, and 73% of their respective PM_2.5_, respectively. This suggests that the contribution of carbonaceous aerosols in the heating season to the urban atmospheric particulates is relatively high. The TCA concentration was especially high in the Langfang atmosphere, which may be because the sampling site in this area was located near residential areas, and heating in winter is mainly achieved by coal burning or household biomass combustion.

It can be seen from the Figure 2 that the air pollution in Langfang was the most serious, and the content of carbonaceous aerosol in PM_2.5_ was also the highest among the three cities. The pollution concentration of PM_2.5_ in Tianjin was higher than that in Beijing, but the carbonaceous aerosol content in PM_2.5_ was the lowest of the three cities.

### 3.2. Correlation Analysis between OC and EC

In the study of atmospheric carbonaceous aerosol components, correlation analysis is usually used to determine whether the pollution sources of OC and EC are consistent [24,25]. In this analysis, OC was considered to be mainly composed of POC emitted directly from the combustion of carbonaceous substances and SOC produced by photochemical reactions in the atmosphere. The EC was relatively stable, mainly emitted from a primary emission source with incomplete combustion of carbon materials [26,27]. When the fitting degree of OC and EC was good, we believe that the sources and their diffusion processes of OC and EC in atmospheric PM_2.5_ were similar [28].

**Figure 3:**
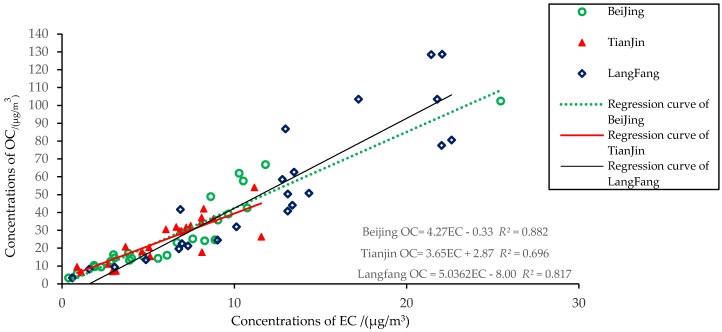
Correlations between organic carbon (OC) and elemental carbon (EC) in winter for the three cities.

The correlation of OC and EC in the heating period in Beijing, Tianjin, and Langfang was studied with the parameter correlation analysis method [29]. The results showed that there was significant correlation at the 0.01 confidence level between OC and EC in the three cities (bilateral). The Pearson moment correlation coefficient (*r*) values were 0.939 for Beijing, 0.834 for Tianjin, and 0.904 for Langfang. Among these, the fitting degree (*R*^2^) of OC and EC between Beijing and Langfang was relatively high and the correlation was strong, indicating that the sources of OC and EC in the atmosphere were similar, but the fitting degree of OC and EC in Tianjin was relatively weak and the source was more complex. The results of the linear regression analysis of the mass concentration of OC and EC in three cities are shown in Figure 3, which shows that the decisive coefficients decreased in the order Beijing > Langfang > Tianjin. This indicates that the source of the Beijing OC and EC had good similarity, but the similarity of Tianjin was poor. In general, the higher the slope of the regression equation, the higher the content of the primary emission sources of OC [5,7]. The highest slope of the three cities was Langfang, which was greatly affected by the primary emission source.

Research shows that throughout the country, the main sources of carbonaceous organic matter in the atmosphere consist of three sources: motor vehicle exhaust emissions, coal burning, and biomass combustion [30,31]. Based on the analysis of the differences of atmospheric carbon-bearing aerosols in the three cities during the sampling period, the main contribution sources in Beijing may have come from motor vehicle exhaust, whereas those in Langfang were coal and biomass combustion, and Tianjin may have been affected by soil and construction dust emissions in addition to coal and motor vehicle exhaust [19].

### 3.3. OC and EC Ratio Analysis and SOC Estimation

To further explore the main sources of OC and EC in each city, the ratio of OC and EC could indicate the main sources of pollution and whether there was SOC generation [20]. Some studies have shown that an OC/EC ratio greater than 2 indicates SOC formation [32,33], whereas when the ratio is between 1.0 and 4.2 [34,35], the exhaust emission of diesel and gasoline motor vehicles is the main source. An OC/EC ratio of 2.5–10.5 indicates coal combustion [36], 3.8–13.2 indicates biomass combustion [37], and 32.9–81.6 indicates catering cooking emissions [38].

**Figure 4:**
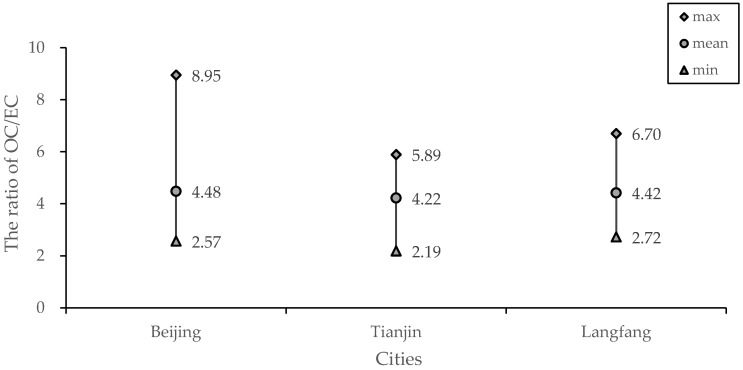
The maximum, mean, and minimum OC/EC ratio values in the three cities.

The ranges of OC/EC ratios at Beijing, Langfang, and Tianjin at the sampling sites were 2.6–9.0, 2.7–6.7, and 2.2–5.9, respectively, and OC/EC mean concentration ratios were 4.88, 4.42, and 4.22, respectively (Figure 4). It can be seen that there was SOC formation in the carbonaceous aerosols of atmospheric PM_2.5_ in the three cities, and the types of major pollution sources could be attributed to compound pollution under the combined effects of vehicle exhaust emissions, coal combustion, and biomass combustion. The sampling points of Beijing and Tianjin were set on university campuses, of which the Beijing sampling point is located 5 m beside the main road of the West Third Ring Road, and the Tianjin sampling point was located on the teaching building roof and was less than 10 m from the east ring road. Therefore, the contribution of motor vehicle exhaust emissions and dust from the road to the two sites may have been considerable. Given that the Langfang site was located in the open space near the residential area of Xianghe County, the burning of coal and biomass by individual residents in winter should have been the most important pollution source in this area. The cooking pollution produced by the collective food supply on the campus was a non-negligible source of atmospheric pollution. The Langfang sampling point was also close to a residential area, so the cooking pollution source contributed considerably to air pollution at this point. Therefore, all three sites would have been affected by cooking pollution sources to a certain extent. In general, based on the range of OC/EC values, it could be concluded that the main sources of pollution in the three cities are the burning of motor vehicle exhaust and heating materials.

The OC/EC ratio results were greater than 2 (4.48, 4.42, and 4.22 for Beijing, Langfang, and Tianjin, respectively), indicating that SOC was produced in the atmospheric carbonaceous aerosols for all three cities [32]. Castro et al. [39] found that because EC mainly comes from the emission of incomplete combustion of carbonaceous substances with stable chemical properties, there was a certain correlation between the EC and the POC produced by primary combustion. Assuming that the value of OC/EC is small, the effect of SOC can be excluded, and the ratio is more stable [8]. Thus, the minimum ratio of OC and EC can be used to quantitatively estimate the size of SOC [32,40]. The empirical formulae are as follows:

POC = EC × (OC/EC)_min_ (1)

SOC = OC − POC(2)

where (OC/EC)_min_ is the minimum value of OC/EC during the sampling period, POC is the estimated primary organic carbon concentration, and SOC is a quantitative estimate of the secondary organic carbon concentration. All units are μg/m^3^. The calculation results are shown in Table 1.

The SOC content in atmospheric PM_2.5_ carbonaceous aerosols in Langfang was relatively high with an average mass concentration of 19.51 μg/m^3^, which accounted for 37% of the OC mass concentration. The average SOC concentration of Beijing and Tianjin was similar, accounting for 38% and 42% of OC, respectively. Because of the frequent snowfall, high average urban humidity (60.6–65.3%), and low average wind speed (1.3–1.7 m/s), the area was prone to reverse temperature inversion in winter, and the continuous accumulation of pollutants in the atmosphere. Therefore, the SOC content in the atmospheric PM_2.5_ carbon aerosol in the Beijing–Tianjin–Langfang region was high. The average mass concentration of SOC in Tianjin accounted for a relatively high proportion of OC, indicating that the source of pollutants in both OC and EC were quite different. This result was in good agreement with the final results of the correlation analysis of OC and EC.

### 3.4. Carbon Component Source Analysis

To further confirm the above speculations, a principal component analysis and a mathematical statistical analysis were used to conduct in-depth analyses of carbon component characteristics in atmospheric PM_2.5_. The thermal optical reflection method with a stepwise temperature increase was used to analyze the carbonaceous components in PM_2.5_, and eight carbonaceous components were produced (OC1, OC2, OC3, OC4, EC1, EC2, EC3, and OP). In general, there were great differences in the carbonaceous components of carbonaceous aerosols emitted to the atmosphere by different pollution sources [6,41,42]. Therefore, the different sources of urban air pollution could be analyzed by analyzing the measured data for the eight carbonaceous components. The source spectrum test [6,43,44] showed that OC1 and OP were the main components of biomass combustion [6]; OC2 released by coal combustion was abundant [45]; and OC2, OC3, OC4, OP, and EC1 were high in gasoline vehicle exhaust [33]. Of these, the EC1 content was the highest, while EC2 and EC3 were mainly associated with the exhaust emissions of diesel vehicles [43], and the definition of OC3 and OC4 remains rather vague. Zhang et al. [46] suggested that a relatively high OC3 and OC4 content indicates a high contribution of the road dust pollution source. Ma et al. [47] suggested that OC3 and OC4 are the main components of road dust and coal combustion as well as the main components of gasoline vehicle exhaust. Therefore, we suggest that it is most suitable to identify the pollution source by combining the other high-load components with the analysis of OC3 and OC4.

Principal component analysis was used to analyze the concentration of the eight components in the atmospheric carbonaceous aerosols in Beijing, Tianjin, and Langfang according to the selection criteria of characteristic factors, that is, the characteristic value was greater than 1.0, and the cumulative variance is greater than 80%. Two main components were extracted from Beijing and Langfang, and three main components were extracted from Tianjin. Among them, two factors in Beijing explained 86.7% of the carbon component, three factors in Tianjin explained 87.1%, and two factors in Langfang explained 80.1%. After the maximum tolerance rotation, the main component factor load matrix is shown in the Table 2.

Table 2 shows that the main components of Beijing factor 1 were OC2, EC1, OC3, OP, and OC1, with factor loads of 0.996, 0.989, 0.968, 0.958, and 0.951, respectively, explaining 62.1% of all carbonaceous components. This indicated that the main sources of pollution in the Beijing samples were coal combustion, gasoline vehicle exhaust emission, and biomass combustion. The main components of factor 2 were EC2 and EC3, which together explained 24.6% of all carbonaceous components, indicating that the exhaust emissions from diesel vehicles were relatively high. The components with the higher load of factor 1 in Tianjin were OC2, EC1, OC1, OC3, and OP, whereas EC2 and EC3 had the highest load in factor 2, explaining 57.6% and 15.4% of all carbonaceous components, respectively. This indicated that the main pollution sources in the Tianjin samples were coal burning, motor vehicle tail gas and biomass combustion. In factor 3, the main components were EC2, OC3, and OC4, which indicated that diesel vehicles and road dusts contributed substantially to the pollution. In factor 1 for Langfang, the carbonaceous components with the higher loads were consistent with those of Beijing and Tianjin, and the pollution sources were also coal burning, gasoline vehicle exhaust emissions, and biomass burning. In factor 2, EC2, EC3, and OC4 showed the highest loads, indicating a high contribution of motor vehicle exhaust in Langfang.

**Figure 5:**
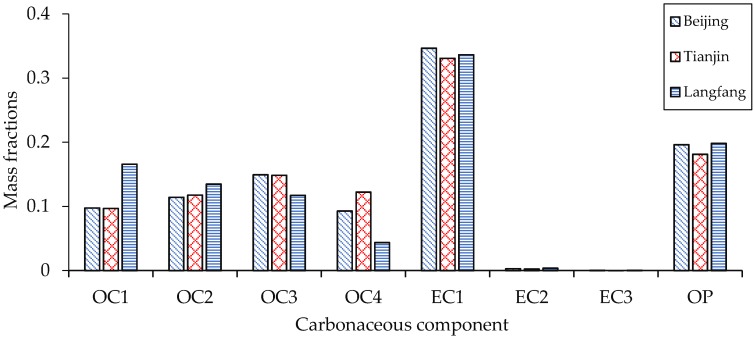
Mass fractions of eight carbonaceous components in Beijing, Tianjin, and Langfang.

In order to further analyze the proportion of the main pollution sources in the three cities, the contents of the eight carbon components were statistically analyzed (Figure 5). The three cities showed roughly the same characteristics with slight differences. Overall, the percentages of EC1 and OP in the three urban carbon components were significantly higher than those in other components, but those of EC2 and EC3 were lower, indicating that the main sources of pollution in Beijing, Tianjin, and Langfang were gasoline vehicle exhaust emissions, while diesel vehicle exhaust contributed less to carbonaceous aerosol in the air. In terms of the differences in carbon components among the three cities, the proportions of various carbon components in PM_2.5_ samples from Beijing and Tianjin were roughly the same, and the composition of atmospheric pollution sources were similar. Among the carbon components, EC1 and OP were higher in Beijing than in Tianjin, and the percentage of OC4 in Tianjin was higher than in Beijing. The proportions of OC1, OC2, OC3, and EC2 were basically the same in the two cities, which showed that the impact of coal-fired and biomass combustion on the two cities was relatively small, and PM_2.5_ in Beijing was dominated by gasoline vehicle emissions. In contrast with Beijing and Langfang, the contribution of the road dust source in Tianjin was too large to be ignored. The carbon component characteristics of Langfang were clearly different from those of Beijing and Tianjin. The proportions of OC1, OC2, and OP in PM_2.5_ samples from Langfang were higher than those in the other two cities, indicating that the contribution of biomass burning and coal burning in Langfang to carbonaceous aerosol in the atmosphere was still high in 2016.

The principal component analysis and statistical analysis of the eight carbon components showed that in the Beijing–Tianjin–Langfang area, the main pollution sources of carbonaceous aerosols in the atmospheric PM_2.5_ were from the exhaust emission of gasoline vehicles. Moreover, the types of pollution sources in each city were slightly different; Tianjin road dust pollution was more serious than the other two cities, and the contribution of biomass burning and coal burning in Langfang was the most prominent of the three cities.

## 4. Conclusions

During the heating period in 2016, the atmospheric PM_2.5_ pollution in Beijing, Tianjin, and Langfang was serious, with average mass concentrations of 113.13 ± 93.03, 126.87 ± 79.10, and 132.1 ± 95.52 µg/m^3^, respectively, which is far higher than the second-level daily average standards limit set by the state. Carbonaceous aerosol accounted for 31%, 25%, and 49% of PM_2.5_ concentration in Beijing, Tianjin, and Langfang, respectively, and it was the main component of fine particles in the air of the three cities, among which the average mass concentrations of OC were 27.93 ± 23.35, 25.27 ± 12.43, and 52.75 ± 37.97 µg/m^3^, respectively. The average EC concentrations were 6.61 ± 5.13, 6.14 ± 2.84, and 12.06 ± 6.81 µg/m^3^, respectively, which showed the spatial variation of Langfang > Beijing > Tianjin. Thus, the atmospheric carbon aerosol pollution in Langfang was more dangerous than that in Beijing and Tianjin.

The Pearson moment correlation coefficient (*r*) of OC and EC in the atmosphere of Beijing, Tianjin, and Langfang were 0.939, 0.834, and 0.904 respectively, which showed a strong correlation. This indicated that the sources of carbonaceous aerosols in urban air were relatively consistent. The correlation of Tianjin was relatively weak and the source of pollution was relatively complex. According to the finding of the OC/EC ratios of about 4, SOC was generated in all three sampling points, and the source of the pollution was initially determined to be the composite pollution of motor vehicle exhaust, and coal and biomass combustion, among which SOC accounted for 42% > 38% > 37% of OC mass concentration in Tianjin, Beijing, and Langfang, respectively. The SOC content in carbonaceous aerosol of PM_2.5_ of Beijing, Tianjin, and Langfang was high during sampling, which was related to the frequent snowfall, high humidity, low temperature, and wind speed in winter, which allowed pollutants to easily and continuously accumulate in the atmosphere.

In this study, a principal component analysis and mathematical statistics of the eight carbon components of PM_2.5_ at the sampling sites allowed for a deep assessment of the similarities and differences in the sources of the three urban air pollution. The major sources of carbonaceous aerosols in atmospheric PM_2.5_ were similar in Beijing, Tianjin, and Langfang, which were mainly gasoline vehicle exhaust emissions, and the contribution of coal combustion and biomass combustion has gradually decreased in recent years.

## Figures and Tables

**Table 1:** Calculated OC/EC ratios and SOC concentrations.

Sites	SOC (μg/m^3^)	SOC/OC (%)	OC/EC
Beijing	10.73	38	4.48 ± 1.45
Tianjin	10.71	42	4.42 ± 1.77
Langfang	19.51	37	4.22 ± 1.29

**Table 2:** Principal component analysis results for the eight carbon fractions in Beijing, Tianjin, and Langfang.

Carbonaceous Components	Beijing		Tianjin			Langfang	
	Factor 1	Factor 2	Factor 1	Factor 2	Factor 3	Factor 1	Factor 2
OC1	0.951	−0.123	0.946	−0.074	−0.098	0.931	−0.234
OC2	0.996	0.04	0.985	−0.073	−0.058	0.965	−0.188
OC3	0.968	0.168	0.856	−0.063	0.313	0.973	−0.136
OC4	0.432	0.065	0.371	−0.585	0.552	0.483	0.604
EC1	0.989	−0.061	0.951	0.023	−0.241	0.958	−0.142
EC2	0.092	0.98	0.159	0.52	0.782	0.549	0.554
EC3	−0.087	0.976	0.293	0.769	−0.066	0.5	0.462
OP	0.958	−0.058	0.924	0.09	−0.215	0.958	−0.167
Contribution rate of variance (%)	62.104	24.62	57.559	15.355	14.195	67.056	13.019
Contribution rate of accumulated variance (%)	62.104	86.724	57.559	72.914	87.109	67.056	80.075
Characteristic valve	4.968	1.97	4.605	1.228	1.136	5.365	1.041
Sources of pollutant	coal combustion, gasoline vehicle exhaust emission, and biomass combustion	diesel vehicle exhaust emission	coal combustion, gasoline vehicle exhaust emission, and biomass combustion	diesel vehicle exhaust emission	diesel vehicle exhaust emission and road dusts	coal combustion, gasoline vehicle exhaust emission, and biomass combustion	diesel vehicle exhaust emission
